# Integrated Role of Nonpharmacological Interventions for Rehabilitation of Individuals with Musculoskeletal Disorders

**DOI:** 10.1155/2020/9493623

**Published:** 2020-06-22

**Authors:** Mario Bernardo-Filho, Borja Sañudo, Adérito Seixas, Danúbia Sá-Caputo, Redha Taiar

**Affiliations:** ^1^Universidade do Estado do Rio de Janeiro, Rio de Janeiro, Brazil; ^2^Universidad de Sevilha, Sevilla, Spain; ^3^Escola Superior de Saúde, Universidade Fernando Pessoa, Porto, Portugal; ^4^Université de Reims Champagne Ardenne, France

Nonpharmacological interventions (NPI) include any treatment without drug treatment without medication such as physical activity and psychosocial interventions (speech-based therapies). These methods have a preventive or therapeutic action and aim to prevent, treat, or cure health problems. It takes the form of a product, method, and program or service whose content must be known by the user, and it is related to biological mechanisms and/or psychological processes. Among them, various technical procedures related to (i) physiotherapy (hand manipulations, electromagnetic radiations, and electrical and ultrasound sources), ((iii) assistive devices, (iv) psychotherapy and behavioral actions (habits in general and nutrition), (v) occupational therapy, (vi) speech and language therapy, and (vii) complementary and alternative medicine [1]. Moreover, physical exercises and vibratory therapy such as segmental and whole-body vibration exercises might be included among the NPI. In fact, various populations including the elderly with different clinical disorders have been submitted to these interventions.

It is important to highlight that nonpharmacological and pharmacological therapies are complementary on the management of the elderly with clinical conditions, often with multimorbidity. It is widely acknowledged that NPI, including surgery, can be effective and sometimes more effective than pharmacological therapy in the treatment of several common, chronic, and undesirable conditions [2, 3] Indeed, [4] consider that NPI in older people can be just as important as pharmacological therapies to treat chronic conditions. It is suggested that ageing populations would require more and more relief from chronic pain and disability and that the prevalence of musculoskeletal disorders (MSDs) will continue to rise [5, 6]. Moreover, MSDs consume a large amount of health and social resources and are a major cause of disability in both low- and high-income countries [7].

MSDs are undesirable multifactorial clinical conditions affecting different human body parts and are the leading cause of years lived with disability in the world affecting children, working age population, and elderly. Although not fatal, these conditions have a high prevalence and significant impact on daily living activities by limiting and restricting the participation of individuals affecting them and society. In addition, individuals with several diseases such as spinal cord injury, cerebral palsy, and stroke are more prone to develop MSDs. Otherwise, there is a widespread underuse of nonpharmacological therapies on the management of chronic diseases and associated clinical conditions of the elderly. Considering that the prevalence of these conditions is expected to increase in the coming years due to ageing, rising levels of obesity, and physical inactivity, there is a clear demand in research focusing on the rehabilitation of MSDs.

Putting together the previous rationale, the challenges and reflections in organizing this special issue, we thank the Hindawi publisher for the confidence. A special thanks to all authors that contributed in this special issue of the journal *Biomedical Research International* entitled “Integrated Role of Nonpharmacological Interventions for Rehabilitation of Individuals with Musculoskeletal Disorders.” The authors tried to bring a useful issue involving the proper use of NPI for the rehabilitation of individuals having musculoskeletal disorders. They contributed by giving scientific evidence and disseminated the knowledge about the benefits and the plurality of NPI and the management of the MSDs. The aim was to provide a multidisciplinary discussion forum covering all rehabilitation professions regarding the integrated role of NPI in the aim to reduce the burden of individuals living with MSDs. The readers will find scientific information about the integrated role of NPI for MSDs in the elderly, sports, and special populations (e.g., pregnant women, cancer patients, and others) and strategies to avoid and manage musculoskeletal disorders in the workplace and prevention of MSDs across all lifespans and settings.

*Mario Bernardo-Filho* *Borja Sañudo* *Adérito Seixas* *Danúbia Sá-Caputo* *Redha Taiar*
